# Evaluation of an HIV-specific rapid response service for community-based organisations in Ontario, Canada

**DOI:** 10.1186/s12961-019-0476-4

**Published:** 2019-08-14

**Authors:** Michelle Camilleri, David Gogolishvili, Danielle L. Giliauskas, Jason Globerman, Michael Wilson

**Affiliations:** 1 The Ontario HIV Treatment Network, 1300 Yonge Street, Suite 600, Toronto, Ontario M4T 1X3 Canada; 2 Department of Health Research Methods, Evidence and Impact, McMaster Health Forum, 1280 Main St West, MML-417, Hamilton, Ontario L8S 4L6 Canada

**Keywords:** rapid synthesis, rapid review, HIV, community organisations, AIDS service organisations

## Abstract

**Background:**

To support AIDS service organisations and other community-based organisations’ use of research evidence to inform HIV-related programmes, services and policies, the Ontario HIV Treatment Network (OHTN) developed a Rapid Response Service. The final product of the rapid response process at the OHTN, which is more streamlined than that of traditional systematic reviews, consists of a detailed report answering questions regarding an HIV-specific issue and how the findings apply within the local context. In 2016, the OHTN conducted an evaluation to assess the effectiveness of its Rapid Response Service. This article reports on the development of this service as well as the results of the evaluation.

**Methods:**

All rapid responses published between January 1, 2009, and September 30, 2016, by the OHTN (*n* = 102) were analysed using univariate analyses. Frequency distributions were determined for the following variables for each rapid response: populations observed, topics covered, requestor affiliations and number of downloads from the OHTN’s website. Requestors of rapid responses were also interviewed regarding perceived helpfulness and utility of the service and final products, and suggestions for changes to the service. Six-month follow-up interviews were conducted to determine how affiliated organisations used the evidence from the rapid response they requested.

**Results:**

The 102 rapid responses published covered 14 different populations of interest. Topics covered included the HIV prevention, engagement and care cascade, determinants of health, syndemics, and comorbidities. Requestor affiliations consisted of AIDS service organisations, government agencies and policy-makers, non-HIV-focused community-based organisations, and hospitals, universities or health centres. Requestors perceived most aspects of the Rapid Response Service as very helpful and most frequently suggested that the rapid responses should provide recommendations. Follow-up interviews regarding the impact of rapid responses show that rapid responses have been used to assist organisations in numerous activities.

**Conclusions:**

Organisations that have used the OHTN’s Rapid Response Service describe it as a valuable service useful for the development of programmes and policies. Improvements in capacity-building efforts may increase its utility. Describing the findings of this evaluation may serve as a reference for similar programmes to increase the use of research evidence among public health decision-makers.

**Electronic supplementary material:**

The online version of this article (10.1186/s12961-019-0476-4) contains supplementary material, which is available to authorized users.

## Background

The use of research evidence to inform policy and practice has been widely accepted among public health professionals [1–6]. However, public health organisations often need evidence synthesised within a timeframe that does not allow for a full systematic review. For this reason, decision-makers have begun to rely on rapid reviews that synthesise research evidence within short periods of time [7, 8].

Typically, rapid reviews are completed within 1–12 months and range from annotated bibliographies, reference lists and abstract summaries to rapid systematic reviews and full health technology assessments [9, 10]. The main goal of most rapid reviews is to inform policy and programme development and decision-making [9, 11, 12] by providing evidence that has been contextualised to a specific health system setting in response to specific issues [13]. There is evidence showing that rapid reviews may improve the clarity and accessibility of research evidence for decision-makers in clinical guideline development [14] as well as be of use for directing future research [15] and for policy decision-making [16, 17]. Yet, as the use of rapid reviews by public health decision-makers grows, there is increasing interest in investigating how and by whom rapid reviews are being used and produced to share best practices and lessons learned [9, 18, 19]. There also remains a need for evaluations of interventions to facilitate the use of research in health policy and programmes, and their potential influence on decision-making [16].

Initiatives designed to promote the use of research evidence among health system and policy decision-makers has steadily increased [4, 5, 16, 17, 20–22]; however, few ventures have been established to support the use of research evidence within community-based organisations (CBOs). Efforts to evaluate the effectiveness of using rapid reviews to inform services and programmes within the community context are even more limited [4]. CBOs and other non-profit and non-governmental groups play a vital role within health systems. CBOs provide services and programmes directly to community members and often support marginalised groups and individuals who may not be comfortable accessing mainstream healthcare services [4]. Many CBOs now mandate the use and production of research evidence to inform their programmes and services. However, this presents CBOs with a number of challenges, since many lack the funding, time, staff and expertise necessary to acquire and assess the literature [4, 23].

In the context of HIV-related public health initiatives in Canada, community actions and programmes have contributed substantially since the beginning of the epidemic [24]. CBOs have a particularly important role in addressing the HIV/AIDS epidemic in the province of Ontario, as it is home to the highest number and proportion of people living with HIV in the country [25]. Community-based HIV prevention programmes have had a great impact on reducing HIV infections as well as healthcare system costs [26], and there is a strong network of over 40 HIV/AIDS-focused CBOs across the province that work towards ending the local HIV epidemic [27]. However, results from an assessment of 25 HIV/AIDS-focused CBOs across Ontario (providing HIV/AIDS services to approximately 32,000 clients per year) still found that capacity to acquire, assess, adapt and apply research evidence was low across these organisations [23]. Therefore, researchers recommended that capacity-building efforts be developed and evaluated in order to facilitate the use of research evidence among HIV/AIDS-focused CBOs to support their use of research evidence to inform programme planning and service delivery [23].

Growing from this research, the Ontario HIV Treatment Network (OHTN) – a non-profit collaborative network aiming to improve the health and lives of people living with and at risk of HIV by using data and evidence to drive change – began to address these challenges by developing a Rapid Response Service in 2009. The OHTN’s Rapid Response Service works primarily with CBOs, both HIV-focused (i.e. AIDS Service Organisations (ASOs)) and non-HIV-focused, that lack the capacity and resources to acquire and assess research evidence. It also provides services to policy-makers (such as provincial health authorities), healthcare providers and academic researchers. With respect to assessing the impact of health research, the literature has identified rapid response programmes as a key mechanism to facilitate what is known as ‘user-pull’ or decision-makers’ efforts to identify research, particularly where a crucial gap in knowledge exists [5, 28, 29]. Efforts to facilitate ‘user-pull’ are thus provided by researchers to make relevant, high quality research evidence available to decision-makers in a timely manner and a useable format [5, 28]. The goal of the OHTN’s Rapid Response Service is to synthesise research evidence to support evidence-informed policies, programmes and services. Globally, the OHTN’s Rapid Response Service is the only service of its kind that we know of which focuses exclusively on HIV and sexual health.

The rapid response process at the OHTN was developed with a commitment to respond to questions from ASOs and other CBOs by systematically and transparently identifying and synthesising relevant research evidence in days or weeks (as opposed to months or years, as can be the case with traditional systematic reviews [30]). The process is conducted by dedicated Knowledge Synthesis and Rapid Response Service staff at the OHTN, and overseen by the organisational manager responsible for research and synthesis activities. The first step of the process focuses on refining a researchable question with the requestor. Collaboration between knowledge producers and users has been reported as a facilitator to the use of research evidence by policy-makers [31], and this feature of rapid reviews can contribute to their usability, relevance and applicability for decision-making [13]. The next step in this process involves identifying existing systematic reviews to determine whether one or more comprehensive syntheses have already been conducted. Depending on the nature of the question and whether systematic reviews are identified, the scope is iteratively expanded to identify primary research from targeted database searches (typically in Medline and/or PsycInfo) and sources for grey literature (e.g. websites of relevant organisations). However, if a topic is too broad, OHTN staff will work with requestors to narrow the scope of the rapid response, for instance, by limiting the number of questions or outcomes considered, or limiting the literature search dates, which are common mechanisms utilised by researchers to enhance the timeliness of rapid reviews [13]. Following this, at least one staff member screens the search results, and retrieves and extracts key findings from relevant systematic reviews and primary studies. These are used to prepare a brief summary of key findings. If required, a supplementary step is also included in this process, where experts, such as researchers, clinicians or community members with specialised knowledge in a particular topic being reviewed, are consulted to provide additional information.

The final product consists of three to five key messages, an outline of the issue and its importance, a detailed summary of findings, and potential factors related to how the findings apply within the local context. All of this information is presented in plain language and in a format that is compliant with legislation in Ontario, Canada, for ensuring accessibility for people living with disabilities (Accessibility for Ontarians with Disabilities Act, 2005). All completed rapid responses – from 2009 to the present – are housed on a publicly accessible page on the OHTN website [32], which works as another effort to facilitate ‘user-pull’ [5].

In 2016, the OHTN conducted an evaluation to assess the effectiveness of its Rapid Response Service. The evaluation consisted of analysing previously published rapid responses and conducting qualitative interviews with requestors to explore how the rapid responses were used by community organisations and other stakeholders to inform services, programmes or policies, create or improve them, or secure new funding [33].

The goal of this article is to share the development process of the OHTN’s Rapid Response Service, report on the evaluation of the programme and outline potential next steps for the Rapid Response Service to address issues raised through this evaluation by community organisations as well as implications.

## Methods

Wilson et al.’s [5] indicators of success and approaches to measurement for a rapid response programme for health system decision-makers provided the guide upon which this evaluation was framed. Areas where the success of a rapid response programme can be measured, as outlined by Wilson et al. [5], include programme organisation (i.e. whether the programme is organised such that decision-makers are able to make a request and receive a response efficiently), final product (i.e. whether the rapid response met the requestor’s needs), and whether and how the rapid response was used [5]. Suggested approaches to measuring these indicators include surveys or interviews that ask requestors to evaluate key features of the rapid response programme, key features of the rapid response synthesis and what was most and least helpful, and how the rapid response was used (conducted 6 months after the final product is received) [5].

All rapid responses published between January 1, 2009, and September 30, 2016, by the OHTN (*n* = 102) were analysed by the dedicated Knowledge Synthesis and Rapid Response Service staff members using univariate analyses. Each rapid response was categorised within the following variables: ‘populations observed’, ‘topics covered’, ‘requestor affiliations’ and ‘number of downloads from the OHTN website’. Frequency distributions of rapid responses were determined for each respective variable. These variables were chosen to reflect assessment of programme organisation, as described by Wilson et al. [5]. Determining the populations observed and topics covered by rapid responses could give an indication of the success of the programme in answering HIV-related questions. Investigating which organisations requested rapid responses could provide indication of the success in reaching ASOs. The number of downloads of rapid responses could provide an indication of whether posting completed rapid responses publicly on the OHTN website facilitated active efforts to identify research evidence outside the service [29].

In addition to the above, interviews with requestors of rapid responses were conducted by Knowledge Synthesis and Rapid Response Service staff members of the OHTN to determine requestor’s perceptions of the final product, and whether the service met their needs. Requestors were first contacted either by email or telephone to answer 10 interview questions (Additional file 1). The first seven questions focused on requestors’ perceptions of helpfulness related to specific features of the Rapid Response Service, and the service overall, using a seven-point Likert scale (1 = very unhelpful, 7 = very helpful). The remaining three questions were open ended and inquired about the most and least useful aspects of the rapid responses and what the OHTN could do differently. People who requested more than one rapid response during the specified time period were surveyed separately for each request [33]. As this process began in the beginning of 2016, and it was predicted that requestors would have difficulties in recalling specific details of older rapid responses, the researchers decided to contact only those who requested a rapid response between 2014 and 2015 (*n* = 25).

As suggested by Wilson et al. [5], requestors who answered the 10 interview questions were then emailed to request in-depth follow-up qualitative interviews (conducted by an OHTN Knowledge Synthesis and Rapid Response Service staff member via telephone) 6 months later (Additional file 2). Interview questions focused on how affiliated organisations used the evidence from the rapid response they requested to inform service, programme or policy issues, create or improve them, or secure new funding [33]. As interviews were conducted for the purposes of evaluating the Rapid Response Service, ethical review was not required by the Public Health Ontario Ethics Review Board [34].

Interviews were transcribed and numeric case identifiers were assigned to each transcript to ensure anonymity. These transcripts acted as the units of analysis and were reviewed by two researchers. Specifically, a content analysis [35], sometimes referred to as thematic content analysis [36, 37], was conducted to attain a condensed and broad description of requestors experiences, and categorise words and phrases describing these experiences found within the data into specific themes [33, 35]. Firstly, researchers individually read and reviewed transcripts, writing notes and headings to describe and become familiarised with the content. Preliminary coding schemes were thus derived from the responses given. Notes and headings were then collected, and preliminary codes were identified by searching for repetition, similarities and differences as well as patterned responses that recurred within the transcripts [35, 36]. No analysis software was used. Rather, a simple ‘cut and paste’ technique of extracting text directly from transcripts and sorting these quotes into the themes within Excel spreadsheets (with case identifiers of original transcripts for each quote) was used [36]. Abstraction of main and subcategories of similar responses was performed, and these categories were discussed and revised until collectively agreed upon amongst the researchers [35]. Disputes were resolved by a third researcher.

## Results

### Profile of rapid responses produced

Between 2009 and 2016, the number of rapid responses published per year varied from seven to 22 for a total of 102 completed rapid responses (Table 1; Additional file 3). At the time of the evaluation, eight requests were in progress and therefore were not included in the analyses. Frequency distributions were determined for the variables of ‘populations observed’, ‘topics covered’, ‘requestor affiliations’ and ‘number of downloads from the OHTN website’ (see Table 1 for frequency distributions; see Additional file 3 for the characteristics of individual rapid responses). It is important to note that variables such as population and topic were not mutually exclusive, as 25 (24.5%) rapid responses covered more than one population and 39 (38.2%) rapid responses covered more than one topic. However, no rapid response had more than one requestor affiliation [33].

**Table 1:** Distribution of variables covered in rapid responses (RRs) by year published

		Total (%)^a^	2009	2010	2011	2012	2013	2014	2015	2016
	Total number of RRs	102 (100%)	13	22	7	12	12	17	8	11
Affiliation type	AIDS service organisation	50 (49.02%)	4	12	6	5	5	8	5	5
	Government agency	19 (18.63%)	2	3	1	2	3	3	1	4
	Community-based organisation	8 (7.84%)	1	5	–	1	–	–	1	–
	Hospital/University/Health centre	7 (6.86%)	–	1	–	2	1	2	–	1
	Ontario HIV Treatment Network	18 (17.65%)	6	1	–	2	3	4	1	1
Populations of interest	General HIV-positive population	40 (39.22%)	7	6	4	5	6	6	2	4
	General HIV-negative population	8 (7.84%)	1	2	–	2	–	–	–	3
	Men who have sex with men	23 (22.55%)	2	5	–	2	4	5	3	2
	Women	7 (6.86%)	–	5	1	1	–	–	–	–
	People who use drugs	8 (7.84%)	1	2	–	1	1	2	–	1
	Youth	6 (5.88%)	–	2	–	1	–	1	2	–
	Ethnocultural minorities	6 (5.88%)	–	1	–	2	1	1	1	–
	Immigrant/Refugee/Non-status	3 (2.94%)	–	1	–	–	1	1	–	–
	Sex workers	2 (1.96%)	–	1	–	1	–	–	–	–
	Transgender communities	2 (1.96%)	–	1	–	–	–	–	–	1
	Indigenous communities	2 (1.96%)	–	–	1	–	–	–	–	1
	Prisoners	1 (0.98%)	–	–	–	1	–	–	–	–
	Older Adults (> 50 years)	1 (0.98%)	–	–	1	–	–	–	–	–
	Other	21 (20.59%)	4	7	1	1	2	2	2	2
Syndemics	Mental health	11 (10.78%)	4	2	1	1	–	2	–	1
	Substance use	13 (12.75%)	3	1	–	2	1	2	1	3
	Co-infections/Comorbidities	9 (8.82%)	–	2	–	1	–	3	–	3
Determinants of health	Health services	20 (19.61%)	–	1	1	2	3	6	2	5
	Social support	8 (7.84%)	1	2	2	–	–	2	1	–
	Stigma/Discrimination	6 (5.88%)	–	2	–	–	1	2	1	–
	Housing	2 (1.96%)	1	–	1	–	–	–	–	–
	Education	1 (0.98%)	–	–	–	–	–	1	–	–
	Employment	1 (0.98%)	–	–	–	1	–	–	–	–
	Other	10 (9.80%)	–	2	3	2	2	1	–	–
Prevention, engagement and care cascade	Epidemiology	9 (8.82%)	–	3	–	2	1	2	1	–
	Testing	12 (11.76%)	1	3	–	1	2	2	2	1
	Prevention	31 (30.39%)	3	12	–	5	3	5	2	1
	Linkage to care	1 (0.98%)	–	–	–	–	–	–	1	–
	Retention in care	1 (0.98%)	–	–	–	–	–	–	1	–
	Treatment/Adherence	15 (14.71%)	–	2	1	1	3	4	2	2
Number of downloads	200–400	21 (20.59%)	10	6	1	–	–	–	1	3
	401–600	21 (20.59%)	1	6	2	1	1	3	4	3
	601–800	20 (19.61%)	2	6	3	2	–	3	1	3
	801–1000	5 (4.90%)	–	1	1	2	–	1	–	–
	1001–5000	31 (30.39%)	–	3	–	6	11	8	2	1
	>5000	3 (2.94%)	–	–	–	1	–	2	–	–
	Not available	1 (0.98%)	–	–	–	–	–	–	–	1

^a^Totals are with regard to the number of rapid responses published on individual variables. Percentages pertain to the proportion of rapid responses published on a specific variable out of the total number of rapid responses published overall (*n* = 102). It should be noted that, with the exception of ‘the number of downloads’, variables are not mutually exclusive, as 25 (24.5%) rapid responses covered more than one population and 39 (38.2%) covered more than one topic (including syndemics, determinants of health, and the prevention, engagement and care cascade). These totals will not add up to the total number of rapid responses published overall (*n* = 102) and percentages will not add up to 100%

#### Populations observed

Of the 102 rapid responses analysed, 39.2% (*n* = 40) focused generally on people living with HIV, whereas 46 (41%) rapid responses focused on one of the five priority populations specifically outlined in Ontario’s HIV/AIDS Strategy to 2026 [38] (Table 1; Additional file 3). These priority populations are men who have sex with men (*n* = 23; 22.5%), people who use drugs (*n* = 8; 7.8%), women (*n* = 7; 6.9%), African, Caribbean and Black communities (*n* = 2; 1.9%), and Indigenous Peoples (*n* = 2; 1.9%). Populations that the Strategy [38] identifies as ‘at-risk’ or otherwise of interest (due to factors that increase their vulnerability to HIV) were also the focus of a small number of rapid responses. These populations include immigrants, refugees or non-status people, sex workers, transgender individuals, prisoners and older adults.

Additional populations covered by the rapid responses included the general HIV-negative population, youth and ‘other’ populations, including healthcare providers, ASO volunteers, infants, migrant workers, women who have sex with women, and residents of rural communities [33].

#### Topics covered

Many rapid responses covered various steps in the HIV care cascade (Table 1; Additional file 3). The HIV care cascade is a framework modelling the continuum of services necessary for a person living with HIV to ultimately achieve an undetectable viral load. Research has shown that HIV testing and diagnosis, linkage to appropriate health services, access to antiretroviral treatment and support to remain in HIV care are necessary steps through which a person living with HIV must pass in order to reach viral suppression [38, 39]. HIV prevention (e.g. ‘The effectiveness of female condoms for preventing HIV/AIDS and factors that impact uptake’; *n* = 31; 30.4%) and HIV treatment (e.g. ‘Peer-based programmes to support antiretroviral adherence’; *n* = 15; 14.7%) comprised the largest areas of focus of rapid responses, and these were followed by testing (e.g. ‘HIV and sexually transmitted infection testing among Indigenous women and women who inject drugs’; *n* = 12; 11.8%), epidemiology (e.g. ‘HIV prevalence and testing among street-involved youth in Ontario’; *n* = 9; 8.8%), linkage to care (e.g. ‘Transitioning from adolescent to adult care in HIV’; *n* = 1; 0.9%), and retention in care (e.g. ‘Reminder systems for people living with HIV’; *n* = 1; 0.9%) [33].

The Canadian federal government recognises that health is determined by the interaction between individual, organisational, environmental and societal factors [40]. These determinants of health in relation to HIV were also the focus of many rapid responses (Table 1; Additional file 3). In particular, the responses explored the impact of health services (*n* = 20; 19.6%), social support (*n* = 8; 7.8%), and stigma or discrimination (*n* = 6; 5.9%) on HIV-related health outcomes. Other determinants included housing, education, employment, and other topics such as criminalisation, migration/immigration and farm work [33].

The separate and joint effects of two or more concurrent comorbidities or social/structural obstacles (syndemics) within certain populations may exacerbate health outcomes, including HIV prevalence, prognosis and burden of disease [40–42]. Many rapid responses focused on syndemics and comorbidities related to HIV (Table 1; Additional file 3). These comprised substance use, including injection drug use, alcohol, methamphetamine, methadone and tobacco use (*n* = 13; 12.7%); mental health, including anxiety, stress, mood disorders and post-traumatic stress (*n* = 11; 10.7%); and co-infections including hepatitis B and C, syphilis, chlamydia and human papillomavirus (*n* = 9; 8.8%) [33].

#### Requestor affiliations

Thirty-eight organisations (not including the OHTN’s different internal departments) requested rapid responses between 2009 and 2016 (Table 1; Additional file 3). These consisted of ASOs (*n* = 24), government agencies and policy-makers (*n* = 19), non-HIV-focused CBOs (*n* = 8), and hospitals, universities or health centres (*n* = 7) [33].

#### Number of downloads from the OHTN’s website

There was a total of 115,174 downloads of rapid responses overall as of June 30, 2017 (Table 1; Additional file 3). Each individual response had been downloaded between 238 and 10,353 times (mean = 1140). The topics of the five most frequently downloaded rapid responses were as follows: tattooing, piercing, scarification, and acupuncture and their relation to HIV (published 2012, *n* = 10,353); the effectiveness of supervised injection sites (published 2014, *n* = 6562); facilitators and barriers to healthcare for lesbian, gay and bisexual people (published 2014, *n* = 6084); complementary, alternative and traditional medicine in HIV care (published 2013, *n* = 4187); and HIV risk among sex workers (published 2012, *n* = 4013) [33].

### Requestor interviews

Twenty-five requestors whose rapid response requests were completed in 2014 or 2015 were contacted for the purposes of evaluation, of which 24 (96%) requestors responded. The one requestor who could not be contacted was no longer employed with the organisations that had made the requests. No other representatives from this organisation felt they could accurately answer the survey about the rapid responses.

Requestors were ASO staff (*n* = 9), researchers from universities or other institutions (*n* = 3), staff from non-HIV-focused CBOs (*n* = 3), healthcare providers (*n* = 3), policy-makers (*n* = 3), lawyers (*n* = 2) and OHTN staff in collaboration with community organisations (*n* = 1) [33].

**Fig. 1:**
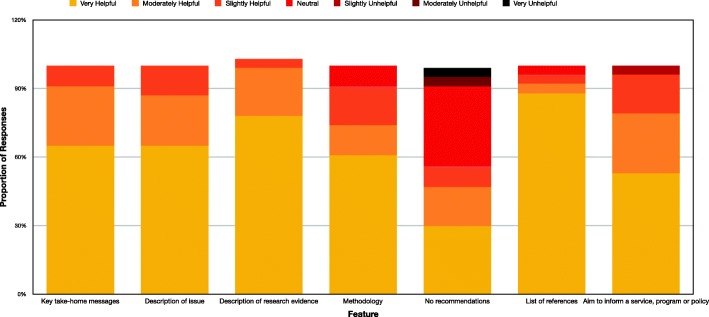
Helpfulness ratings of rapid response features completed by requestors (*n* = 24). Bar graph of the proportion of requestors who gave specific ratings to each section of the rapid response in their initial interview

Helpfulness ratings for each section of the rapid review indicated that, from the perspectives of the requestors, most aspects of the rapid responses were considered useful for their respective organisations. Figure 1 displays the distributions of helpfulness ratings given for each section.

#### Most useful aspects of rapid responses

When asked to identify which particular aspects of the rapid responses they found most useful, the features identified by the most participants were ‘easy to read’ format, overview of the literature and key take-home messages.

The ‘easy-to-read’ format was identified as the most useful aspect by 17% of requestors (*n* = 4). As one requestor stated, the translation of academic literature into lay language can be extremely beneficial, particularly for CBOs. For example, one requestor stated that, “*the synthesis of the literature and having people who have time and ability to do that is helpful, and having them know how to synthesize the data, which is something that community members don’t always have*” [33].

An overview of the literature was also cited as the most useful aspect of rapid responses by 17% of requestors (*n* = 4). In these cases, a lack of organisational capacity with regards to time, resources, access to literature and expertise in research were described as barriers to conducting systematic searches that the rapid response addressed.

Seventeen percent of requestors (*n* = 4) described the ‘key take-home messages’ section of the rapid responses as the most useful aspect, as it proved crucial for the interpretation of the research evidence. Specifically, they referred to this aspect as facilitating quick comprehension of the data for readers who had limited time to conduct this type of research or who lacked research skills. For example, one requestor highlighted that, for them, “*the key take-home messages are good for people who don’t come from a research background*” [33].

Being provided with a list of references was described as most useful by 13% of requestors (*n* = 3). These requestors appreciated that the reference list allowed one to find more information on a given topic, and felt that it was a valuable resource for decision-making, programme development, and manuscript and grant writing. One requestor explained that “*it was great to have the references … We used it as a document to support funding …* [it] *helped us write the background of our paper*” [33].

Two requestors (9%) felt that the ‘what we found’ section of the rapid responses provided a strong background for future research, or offered the appropriate foundation from which programmes and services could be refined: “*The What We Found section provided the information we needed to give consideration to the programming we wanted to develop*” [33].

‘The issue and why it’s important’ section was identified by one participant (4%) as creating a starting point from which to begin their own research, who stated that “*this section helped create a baseline of information … a quick take was needed on where the research was. We needed a starting point and that was what the rapid response was for*” [33].

#### Least useful aspects of rapid responses

Almost two-thirds of requestors (65%; *n* = 15) said that they did not find any aspect of the rapid responses unhelpful. Thirteen percent of requestors (*n* = 3) who did find limitations within the rapid responses stated that the evidence itself was the least useful aspect of the rapid responses. Some of these requestors explained that the evidence was insufficient, either because it did not reveal anything new or because it was not necessarily relevant to their specific contexts. A lack of detail in the information provided was described by 13% (*n* = 3) of requestors, but most conceded that there was a paucity of Canadian research evidence on their respective areas of interest. For example, one participant indicated that “*most of the data cited was American … there was a shortage of Canadian data*” [33]. In addition, one requestor (4%) felt that the lack of a recommendations section was the least useful and one requestor (4%) felt that the responses lacked sufficient information about the search strategy used to find research evidence [33].

#### What can be done differently?

Many requestors (43%; *n* = 10), when asked if there was anything the Rapid Response Service could do differently, responded with ‘No’. Those who did feel that there was a need for improvement, suggested that the Rapid Response Service should provide recommendations (13%; *n* = 3), decrease the time necessary to produce the rapid response (9%; *n* = 2), place more focus specifically on the requestor’s question (9%; *n* = 2), provide a data extraction table with all references (4%; *n* = 1), expand the ‘key take-home messages’ section (4%; *n* = 1), and allow the reviews to answer multiple questions (4%; *n* = 1) [33].

### Follow-up interviews

We contacted each of 24 requestors who completed the initial qualitative survey by email for 6-month follow-up telephone interviews and 75% (*n* = 18) responded. These requestors were ASO staff (*n* = 8), researchers from universities or other institutions (*n* = 3), staff from non-HIV-focused CBOs (*n* = 2), policy-makers (*n* = 2), lawyers (*n* = 2) and healthcare providers (*n* = 1). Of the six requestors who did not respond, affiliations were as follows: CBO staff (*n* = 1), OHTN staff (*n* = 1), healthcare provider (*n* = 2), policy-maker (*n* = 1) and ASO staff (*n* = 1) [33].

#### How could the Rapid Response Service be improved?

Of the requestors who offered suggestions about improving the Rapid Response Service (*n* = 14), some thought the communication between OHTN staff and requestors could be improved. Some respondents felt they were unaware of the ‘commitment’ that the rapid response process entailed with regards to developing a research question and would have liked to have been more prepared for the process as a whole. Others expressed a desire to be more involved in the writing of the rapid response or to have had more discussions with OHTN staff during the writing process. Some felt that their organisations lacked internal research capacity and required further resources or communication with the OHTN for community members to conduct their own searches in the future. For example, two participants shared the following feedback:

“*It may be helpful to have some resources on how community members and others without the expertise can do their own ‘quick’ research, some capacity building*” [33].

“*It might be nice for the OHTN to have a conversation once the rapid response is submitted with the requestor for capacity building purposes. A dialogue might help the translation of the rapid response (such as a grant proposal) be as true to the evidence as possible*” [33].

While the overall impression from the requestors’ responses was that the Rapid Response Service was successful in presenting available research on a topic, some felt that additional Canadian data would be useful for informing their programmes, services and policies. These requestors conceded, however, that this lack of data did not reflect the quality of the rapid responses, but rather highlighted a need for more Canadian HIV-related research. One requestor felt that the rapid responses could be updated to keep up with rapidly emerging research*:* “*As good as the rapid response was, a lot has been coming out since it was completed and the rapid response is now out of date*” [33].

Another requestor explained how the fact that the Rapid Response Service was unable to locate local data on their issue inspired the development of a Canadian study: “*the rapid response mainly cited Australian and U.S. data so a Canadian study has now been developed*” [33].

Many requestors also suggested that the rapid responses should be distributed more widely*.* One requestor specifically stated that, “*these rapid responses are very valuable, but their value is diminished in that they only go as far as a website. I would like to see them go further*” [33], suggesting that additional dissemination and knowledge exchange efforts could potentially increase the impact of the service.

#### The impact of the Rapid Response Service

As outlined previously, many requestors noted that the rapid response was crucial for programme or service development – all requestors gave at least one example of a project or service that was developed with help from the rapid responses. Of the examples given, four referred to new projects that the rapid response assisted in creating. These included needs assessments, evaluations, interviews, pilot studies and community-based initiatives [33].

Two requestors used the rapid response as a guide to ensure that existing programmes and services were in line with current research evidence: “*The rapid response was valuable in providing evidence that what we had already stated was correct*” [33].

For three requestors, the rapid response was used to advocate for funding. These requestors stated that the rapid response had provided the necessary evidence to seek funding and some had been successful in these pursuits as a result: “*We were looking for something to advocate for the funding of skills development to better address marginalized populations, and this rapid response was the piece that allowed our AIDS service organization to tangibly enter the discussion*” [33].

Other uses of the rapid responses that were discussed by requestors included assisting in human rights advocacy, developing an organisational website, creating conference materials and improving inter-agency programming to collaborate with partnering organisations [33].

## Discussion

### Principal findings

The analysis of rapid responses shows that roughly 14 new rapid responses are published each year by our programme, the majority of which were requested by ASOs. Rapid responses covered priority populations identified in Ontario’s HIV Strategy to 2026, HIV-related syndemics and comorbidities, social determinants of health, and all aspects of the HIV prevention, engagement and care cascade. With respect to the success of the programme organisation of the Rapid Response Service, it appears as though the service is indeed being accessed by HIV/AIDS-focused CBOs and addressing their HIV/AIDS-related research questions.

Helpfulness ratings imply a high level of satisfaction with the Rapid Response Service as well as its final products. Requestors found most aspects of the service very helpful, particularly the ‘What we found’ section, the list of references and the key take-home messages.

Responses from the ‘Most useful aspects’ section of the survey revealed that reference lists and key take home messages provide foundations from which to expand upon to develop programmes and services. While lack of data was the most frequently mentioned feature in the ‘Least useful aspects’ section, the majority of respondents could not identify any feature.

Similarly, the majority of respondents could not provide any suggestions when asked if there was anything the Rapid Response Service could do differently. The most frequently cited suggestion was to provide recommendations.

The Rapid Response Service appears to effectively support the use of research evidence within HIV/AIDS-focused CBOs and other organisations. Interview responses regarding the impact of rapid responses showed that rapid responses have been used to assist organisations in numerous activities, including funding, programme development, evaluations, pilot studies and improving inter-agency programming – indicating success in this regard.

These findings are comparable to other recent evaluations of similar rapid response services. For example, a recent study conducted by Hartling et al. [15] investigating end-user perspectives on the utility and limitations of rapid products (including rapid responses) found that participants felt that rapid responses were useful for understanding the breadth of existing evidence on new and emerging topics. They also found that rapid responses were viewed as useful ‘interim products’ to catalyse change towards future investigations and decision-making [15]. Much like this investigation, participants in Hartling’s investigation felt that these products should include considerations of clinical significance and specific recommendations for future research [15].

Another recent study exploring Ugandan policy-makers’ experiences with rapid response briefs [43] found that, overall, participants felt these products were valuable. However, frustrations with certain aspects of the rapid responses, such as a lack of recommendations, impeded optimal user experience [43].

Moore et al.’s [17] study examining the use of rapid reviews commissioned through a knowledge brokering programme (Evidence Check) by Australian policy-makers also found that a large proportion of reviews (89%) were used to inform activities. Drawing on previous work by Pelz [44], these activities were categorised as being instrumental (i.e. used directly to solve a specific problem), conceptual (i.e. used to understand a specific issue) or symbolic (i.e. used to justify decisions made) [17]. Policy-makers in this study mainly used rapid reviews in instrumental and conceptual ways, including for determining the details of a policy or programme, identifying priorities for future action or investment, negotiating interjurisdictional decisions, evaluating alternative solutions for problems, and communicating information to stakeholders [17]. The reported uses of rapid responses requested through the OHTN’s Rapid Response Service also reflect instrumental, conceptual and symbolic purposes, as requestors discussed rapid responses as helping to solve specific problems, furthering understanding of issues and justifying existing practices [33].

### Strengths and limitations

This evaluation provides empirical information regarding the utility of a rapid response service for HIV/AIDS-focused CBOs and other organisations. Utilising a mixed methods approach, the evaluation includes profiles of a set of rapid responses conducted (offering insight into the priorities of CBOs in the largest Canadian province), feedback of the key features of the programme, and qualitative interviews to provide insight for whether and how the products were used. As few programmes that support the use of research evidence within the community context have been established [4], we have offered an important contribution to a literature on rapid reviews that is still relatively nascent.

However, some limitations to this evaluation do exist. Firstly, the process entailed contacting previous and current users of the programme. In certain instances, this proved difficult, since some contacts had left their organisation. Interviews were therefore only conducted with people who had requested rapid responses between 2014 and 2016. Insights from requestors who received rapid responses before this time would have been valuable for this evaluation to compare comments as the Service evolved from 2009 to 2016. There is also the possibility of recall bias [45]; due to the time that had passed since some rapid responses were requested, some participants had difficulty recalling exact details about how the rapid response was utilised. It may be useful for the Rapid Response Service to provide feedback surveys immediately following the receipt of rapid responses to avoid this in the future, though this has not yet been implemented at the OHTN.

### Implications

With the increasing demand for rapid reviews by public health decision-makers, there is a growing need for evaluation of interventions designed to promote access to these products, and investigation of whether and how they are being used [9, 16–19]. Describing the process of the OHTN’s Rapid Response Service, as well as results of the assessment of its programme organisation, final products and uses by requestors, contributes to this growing field and offers additional support for the potential value of a rapid response service. More importantly, this evaluation provides lessons to be learned, not only for the future of the OHTN’s service, but for the development of similar programmes. Given that requestors from HIV/AIDS-focused CBOs in Ontario have found this service helpful and have used its products for decision-making, similar programmes may be beneficial for other jurisdictions or sectors that have comparable or lower capacity for research application (as has been historically reported by CBOs in the province) [23].

The information gleaned from our analyses of rapid responses and requestor interviews provides many insights into how the Rapid Response Service can be improved to better inform the service, programme, and policy efforts of CBOs, government agencies and other stakeholders. While the service is being accessed by CBOs, there is a need to extend the reach of the Rapid Response Service. Profiles of rapid responses show that roughly 39% of them focused on the general HIV-positive population and not on any one specific population. These numbers suggest that organisations serving certain populations may not be aware of the OHTN’s Rapid Response Service [33]. Furthermore, frequency distributions of requestor affiliations show that only two organisations (one being the OHTN’s various departments) accounted for almost 30% of requested rapid responses. Another 14 organisations requested a rapid response more than once (between two and seven requests per organisation, accounting for 47% of the total number of requests) [33]. While repeated use of the Rapid Response Service indicates a level of satisfaction with the service, increasing outreach to other organisations would be an important step for the OHTN to take. When decision-makers seek support for the synthesis of research evidence, many utilise internal support services and often work only with external researchers they are familiar with [4, 5, 46]. Hartling’s investigation of user experiences also found that a trusted relationship between the user and producer was perceived to be a critical feature of rapid evidence products [15]. Indeed, many of the requestors who have utilised the Rapid Response Service in our evaluation had had a previously established relationship with the OHTN before submitting a request for a rapid response. The OHTN may benefit from consultations with these longstanding partners to understand and address barriers to engagement with the Rapid Response Service. The Rapid Response Service is promoted on the OHTN’s website; however, increasing communication efforts with organisations that do not currently have a relationship with the OHTN may also be valuable. These partnerships can potentially be achieved through the use of knowledge brokers [4], which have been shown to effectively facilitate the instrumental, conceptual and symbolic use of commissioned rapid review evidence among policy-makers [17].

The Rapid Response Service could also focus on actively promoting awareness of the service by sharing published rapid responses more widely. Facilitating ‘user-pull’ may be improved by prompting partners for research where gaps in literature are identified and building their capacity to access and apply research evidence within their local contexts [47].

The fact that many requestors applied reference lists and key take-home messages to programmes, services and policies, but wanted further recommendations to be provided, demonstrates that requestors are willing to engage with research, but are not always sure how to do this. An explicit decision was made by the OHTN to not include recommendations in the Rapid Response Service, and this decision is similar to those of other rapid evidence services (e.g. [43]). While it may be within the capacity of the Rapid Response Service to provide recommendations, this would require the authors of each synthesis to make judgments based on their personal values and preferences, which are not derived directly from the research evidence. Helping organisations identify areas where rapid responses could be used is one way the OHTN could strengthen capacity-building initiatives [4]. The OHTN maybe also be able to provide requestors with examples of where rapid responses have been used effectively by other organisations to leverage programmes or inform services.

The desire for greater transparency in the research process is evident in responses to the ‘Least useful aspect’ and ‘What the OHTN could do differently’ survey sections. Requestors said they would like rapid responses to describe the search strategies used and data extraction tables; one requestor even suggested that the rapid response should include a chronology of the process from the initial research question to the end product [33]. Transparency has been addressed in the literature on rapid review methodology [12]. One suggestion is that, rather than formalising methodology, researchers should focus on increasing the transparency of the methods used for each review [7, 12, 48] since few rapid reviews address biases or limitations in methodology [7, 49] and there are no standardised methods for rapid reviews [50]. The OHTN could therefore consider offering requestors the option of including search strategies and data extraction tables as appendices to rapid responses. Potentially, requestors could base future research efforts on similar strategies. As two requestors noted that there should be more focus on their specific questions as an area for improvement, communication with requestors regarding the shortcomings of rapid response methodology can also be incorporated into the rapid response process. Reports from end-users of other rapid review services have also expressed that narrowing the scope of requestor’s questions can be problematic in this regard [15]. Explicitly outlining the trade-offs of rapid evidence products compared to full systematic reviews to end-users at the beginning of the process has been recommended [10] to manage expectations as well as to help end-users understand the limitations of streamlined methods [15].

Indeed, increasing communication with requestors during the rapid response process for capacity-building purposes may be another area to improve. Reviews of rapid review programmes have suggested that close relationships and personal contact facilitate the use of knowledge products and the use of research evidence [21, 51]. Requestor interviews also revealed that requestors would find consultations with the OHTN throughout the rapid response process to discuss implications for practice as well as implementation strategies extremely beneficial. One particular study [52] describing the development and assessment of the rapid review process used in the Knowledge to Action research programme at the Ottawa Hospital Research Institute highlights the mutually beneficial effects of ongoing follow-up and collaboration for both knowledge users and researchers [52]. In this case, continuous engagement with knowledge users regarding the utilities of their products led to improvements in meeting the user’s decision and policy-making needs as well as informing the evolution of the Ottawa Hospital Research Institute’s methods [52]. Documentation and evaluation of efforts to engage knowledge users in the knowledge synthesis process of systematic reviews has also been recommended by research to increase their utility and relevance [53]. The OHTN may, therefore, benefit from increasing exchange efforts to work more collaboratively during and following the production of rapid responses [4, 22, 53, 54]. Some requestors also noted a lack of research in relation to their area of interest, which affected the usefulness of the rapid response. Some researchers have suggested that rapid reviews should strongly encourage follow-up research [55]. It may be useful for the OHTN to consider prompting community partners to conduct research in areas where gaps exist in the literature. Notices regarding gaps in research could potentially be included on the OHTN website and in email newsletters. Interview responses regarding the impact of rapid responses show that rapid responses have been used to assist organisations in numerous activities, including funding, programme development, evaluations, pilot studies and improving inter-agency programming. Investigating which capacity-building and knowledge translation strategies are most effective for increasing the use of rapid responses for decision-making [48], furthering our understanding of the types of uses for rapid responses [17], and in-depth consultation with requestors to understand their specific needs [23] may be important next steps for the Rapid Response Service.

This speaks to a growing discussion in healthcare emphasising collaborative approaches (i.e. those that bring together researchers who study societal issues and those who act within them [56]) to knowledge production and use [57, 58], and their potential to generate useful knowledge and broaden research capacity in the process [58]. The nature of researchers and requestors refining a rapid review question together may already facilitate their use, but this may be further facilitated by continuous engagement through the rapid review process [17]. Quality relationships between relevant stakeholders of this research may also foster other collaborative actions [57], which has implications for achieving wider, more sustainable impacts of research [58].

Metrics categorising the uses for rapid reviews among CBOs would be important for other evaluations of rapid response services designed for these organisations. Moore et al.’s study among policy-makers demonstrated that not only did policy-makers use rapid reviews for instrumental, conceptual and symbolic purposes, but also that these products could have multiple types of uses at once, with benefits beyond those for which they were requested [17]. Moore et al. also noted that, as rapid reviews are used to solve problems within a specific policy context, the context itself may be impacted by the solutions generated [17].

Indeed, much focus is being directed by researchers towards whether and how rapid review products are being used to make public health decisions; however, there also remains the enduring need to investigate whether their use has helped to make better decisions. While it is believed that the use of research evidence to inform decision-making (rather than changes in health outcomes) is the most appropriate and easily assessed measure of research impact in public health [29], and it is expected that increases in this use will lead to improved health outcomes [59], the relationship that research evidence has with specific settings and contexts appears to be one that bears further exploration [17]. As the role of CBOs in health system decision-making has been growing in importance around the world [60], continuing to explore the impacts of research on community actions is paramount.

## Conclusions

Organisations that have used the OHTN’s Rapid Response Service describe it as a valuable service useful for the development of programmes and policies. The service must ultimately fit into a larger knowledge translation initiative in order to reach its full potential. Increasing capacity-building efforts, and working more collaboratively with CBOs throughout the rapid response process, may maximise its utility. Future research efforts should also be focused on exploring what facilitates the use of rapid response products as well as the types of activities that they are being used for. In the meantime, outreach to additional CBOs and other organisations should be extended to foster further collaboration. Describing the process and findings of this evaluation provides lessons to be learned for the development of similar programmes that aim to promote the use of research evidence among CBOs and other stakeholders.

## Additional files

Additional file 1: Rapid response evaluation requestor interview questionnaire. Interview questionnaire document given by email or telephone to requestors who requested a rapid response between 2014 and 2015. (DOCX 14 kb)

Additional file 2: Rapid response evaluation 6-month follow-up interview guide. In-depth 6-month follow-up interview document emailed to requestors who completed an initial interview in 2016. (DOCX 12 kb)

Additional file 3: Characteristics of individual rapid responses published by the Ontario HIV Treatment Network between 2009 and 2016. Table of the characteristics of individual rapid responses, including title, year published, population of interest, topics covered and number of downloads as of June 30, 2018. (DOCX 32 kb)

## Data Availability

The data analysed during the current evaluation are not publicly available, but a de-identified version of the datasets is available from the corresponding author upon reasonable request.

## Abbreviations

ASO: AIDS service organisation
CBO: community-based organisation
OHTN: Ontario HIV Treatment Network
