# Supplementary material for: Evaluation of an HIV-specific rapid response service for community-based organisations in Ontario, Canada
Source: Health Res Policy Syst. 2019 Aug 14;17:80. doi: 10.1186/s12961-019-0476-4 (PMC6693275; doi:10.1186/s12961-019-0476-4)
Supplement: Supplementary file 1 — Rapid response evaluation requestor interview questionnaire. Interview questionnaire document given by email or telephone to requestors who requested a rapid response between 2014 and 2015. (DOCX 14 kb) [file 12961_2019_476_MOESM1_ESM.docx]

**Additional File 1. Rapid response evaluation requestor interview questionnaire.**

| **OHTN Rapid Response Service Evaluation**  Through this survey, we are hoping to hear about your experience with the OHTN’s Rapid Response Service. This survey aims to evaluate the impact of this program as way of supporting the use of research evidence by community-based organizations.  Your responses will be anonymized and kept confidential. If you have any questions or concerns about this evaluation or the Rapid Response Service, please contact Jason Globerman at [jgloberman@ohtn.on.ca](mailto:jgloberman@ohtn.on.ca) or 416-642-6486 ext. 2206 | | | | | | | |
| --- | --- | --- | --- | --- | --- | --- | --- |
| **Section A – Views about how the Rapid Response was produced and designed**  In this section we’d like to hear about your views on how helpful the Rapid Response was. Please rate your satisfaction/dissatisfaction, with respect to various components of the review, on a scale of 1-7. | | | | | | | |
| **Question** | **Response options** | | | | | | |
|  | **1** | **2** | **3** | **4** | **5** | **6** | **7** |
| 1. The Rapid Response included a list of key take-home messages. Did you find this helpful? | Very unhelpful | Moderately unhelpful | Slightly unhelpful | Neutral | Slightly helpful | Moderately helpful | Very helpful |
| 2. The Rapid Response described the issue and why it’s important. Did you find this helpful? | Very unhelpful | Moderately unhelpful | Slightly unhelpful | Neutral | Slightly helpful | Moderately helpful | Very helpful |
| 3. The Rapid Response described what we found based on current research evidence about the question and where there are gaps. Did you find this helpful? | Very unhelpful | Moderately unhelpful | Slightly unhelpful | Neutral | Slightly helpful | Moderately helpful | Very helpful |

| 4. The Rapid Response employed a methodologically sound and transparent method to identify, select, and assess available research evidence. Did you find this helpful? | Very unhelpful | Moderately unhelpful | Slightly unhelpful | Neutral | Slightly helpful | Moderately helpful | Very helpful |
| --- | --- | --- | --- | --- | --- | --- | --- |
| 5. The Rapid Response did not conclude with particular recommendations. Did you find this helpful? | Very unhelpful | Moderately unhelpful | Slightly unhelpful | Neutral | Slightly helpful | Moderately helpful | Very helpful |
| 6. The Rapid Response included a list of references for those interested in reading more about a particular topic. Did you find this helpful? | Very unhelpful | Moderately unhelpful | Slightly unhelpful | Neutral | Slightly helpful | Moderately helpful | Very helpful |
| **Section B – Overall assessment of the Rapid Response** | | | | | | | |
| **Question** | **1** | **2** | **3** | **4** | **5** | **6** | **7** |
| 7. The purpose of the Rapid Response was to present the available research evidence to inform a service, program or policy. How well did the Rapid Response achieve its purpose? | Very unhelpful | Moderately unhelpful | Slightly unhelpful | Neutral | Slightly helpful | Moderately helpful | Very helpful |
| **Section C – Views about what can be done better or differently** | | | | | | | |
| **Question** | | | | | | | |
| 8. Please list **one** element of the Rapid Response that you found most useful. | | | | | | | |
| 9. Please list **one** element of the Rapid Response that you found least useful. | | | | | | | |
| 10. Is there anything you would like us to add to the review or do differently? | | | | | | | |

| **Section D – Role and background** |
| --- |
| 11. I am a (please tick (√ ) single most appropriate role category):   \| **Role category** \| **Tick**  **(√)**  **single most appropriate** \| \| --- \| --- \| \| Policymaker (i.e. AIDS Bureau) \|  \| \| AIDS service organization staff person \|  \| \| Community-based organization staff person \|  \| \| Healthcare provider at a hospital, community-health centre, etc. \|  \| \| Researcher in a university or other institution \|  \| \| OHTN staff person \|  \| \| Other (please specify) \|  \| |
| 12. I have extensive training and/or research experience conducting systematic, scoping and/or literature reviews:  YES/NO |
