# Supplementary material for: Evaluation of an HIV-specific rapid response service for community-based organisations in Ontario, Canada
Source: Health Res Policy Syst. 2019 Aug 14;17:80. doi: 10.1186/s12961-019-0476-4 (PMC6693275; doi:10.1186/s12961-019-0476-4)
Supplement: Supplementary file 2 — Rapid response evaluation 6-month follow-up interview guide. In-depth 6-month follow-up interview document emailed to requestors who completed an initial interview in 2016. (DOCX 12 kb) [file 12961_2019_476_MOESM2_ESM.docx]

**Additional File 2. Rapid response evaluation 6-month follow-up interview guide**

| **OHTN Rapid Response Service Evaluation**  Through this interview, we are hoping to hear about your experience with the OHTN’s Rapid Response Service. The aim is to evaluate the impact of this program as way of supporting the use of research evidence by community-based organizations.  Your responses will be anonymized and kept confidential.  These in-depth questions will ask you about your experiences with the Rapid Response Service including whether and how the review we conducted for you was helpful. |
| --- |
| **Section A: Whether and how the Rapid Response was used**  I would now like to ask you a few questions about whether and how you or your agency/institution/organization used the Rapid Response. |
| 13. Could you describe your general impression of the Rapid Response? [provide additional prompts as needed] |
| 14. The purpose of the Rapid Response was to present the available research evidence on a particular topic in order to inform services, programs and/or policies. How well do you think the Rapid Response achieved its purpose? Why?  [provide additional prompts as needed] |
| 15. Based on your reading of the Rapid Response, please describe any elements of the review that you thought were particularly helpful. Why? [provide additional prompts as needed] |
| 16. Based on your reading of the Rapid Response, please describe any elements of the review that you thought could be improved. Why? [provide additional prompts as needed] |
| 17. Please describe any important actions that you or your agency/institution/organization have taken to better inform a service, program or policy issue as a result of what you learned in the Rapid Response. Why?  [provide additional prompts as needed] |
