# Supplementary material for: Evaluation of an HIV-specific rapid response service for community-based organisations in Ontario, Canada
Source: Health Res Policy Syst. 2019 Aug 14;17:80. doi: 10.1186/s12961-019-0476-4 (PMC6693275; doi:10.1186/s12961-019-0476-4)
Supplement: Supplementary file 3 — Characteristics of individual rapid responses published by the Ontario HIV Treatment Network between 2009 and 2016. Table of the characteristics of individual rapid responses, including title, year published, population of interest, topics covered and number of downloads as of June 30, 2018. (DOCX 32 kb) [file 12961_2019_476_MOESM3_ESM.docx]

**Additional File 3. Characteristics of individual rapid responses published by the OHTN between 2009 and 2016**

| **Title** | **Affiliation Type** | **Publication Year** | **Population** | **Syndemics** | **Determinants of Health** | **Cascade** | **Total # of Downloads (as of June 30, 2017)** |
| --- | --- | --- | --- | --- | --- | --- | --- |
| HIV Medication and Depression | ASO | 2009 | General HIV+ | Mental health | - | - | 315 |
| Impact of Methamphetamine and Poppers on Sexual Risk Taking | ASO | 2009 | MSM | Substance use | - | Prevention | 768 |
| HIV Testing Practices | Government Agency | 2009 | Other | - | - | Testing | 316 |
| Languages in Ontario | OHTN | 2009 | Other | - | - |  | 294 |
| Linking Sexual Compulsivity and HIV Transmission | OHTN | 2009 | MSM, General HIV+ population | Mental health | - | Prevention | 320 |
| Mental Health Issues and HIV/AIDS: HIV-specific Screening and Treatment Tools for Mood Disorders | OHTN | 2009 | General HIV+ population | Mental health | - | - | 282 |
| Housing and Harm Reduction | OHTN | 2009 | Drug Users | Substance use | Housing | - | 734 |
| Voluntary Workers in AIDS Service Organizations (ASOs) | OHTN | 2009 | Other | - | - | - | 367 |
| HIV Disclosure | ASO | 2009 | General HIV+ | - | Social support | - | 252 |
| Massage and Recreation Therapy for Reducing Stress | ASO | 2009 | General HIV+ | Mental health | - | - | 379 |
| Does living with HIV/AIDS increase the risk of substance use? | OHTN | 2009 | General HIV+ | Substance use | - | - | 312 |
| H1N1 Vaccine Guidelines for People Living with HIV/AIDS | Government Agency | 2009 | General HIV+ | Co-infections or Co-morbidiities | - | - | 369 |
| Effectiveness and Impact of the Using the Internet for Prevention of HIV/AIDS | CBO | 2009 | Other, General HIV- population | - | - | Prevention | 430 |
| Effectiveness of Female Condoms for preventing HIV/AIDS and Factors that Impact Uptake | ASO | 2010 | Women | - | - | Prevention | 707 |
| Cost-effectiveness of HIV/AIDS front-line services | OHTN | 2010 | Women, MSM, Youth, Drug users | Co-infections or Co-morbidiities | - | Prevention, Testing | 450 |
| Women and HIV/HCV Co-Infection | ASO | 2010 | Women | Co-infections or Co-morbidiities | - | Epidemiology (i.e. HIV incidence/  prevalence), Treatment/  Adherence | 310 |
| Knowledge and Training Needs of Health Professionals Working with People with HIV: With Consideration for Mixed Urban/Rural Care Settings | ASO | 2010 | Other, General HIV+ population | - | - | - | 641 |
| The Use of Facilitated Peer Support Group Model for People Living with HIV | ASO | 2010 | General HIV+ population | - | Social support | - | 1056 |
| HIV Prevention for Women who have Sex with Women | ASO | 2010 | Other | - | - | Prevention | 350 |
| Effectiveness and Cost-Effectiveness of Pre & Post-Exposure Prophylaxis for HIV | Government Agency | 2010 | Women, MSM, Drug users, Other | - | - | Prevention | 1731 |
| Impact of Fetal Alcohol Spectrum Disorder on HIV Risk | ASO | 2010 | Other | Substance use | - | Prevention | 335 |
| Mental Health Status of Women and Families Infected and Affected by HIV/AIDS | ASO | 2010 | Women, Other | Mental health | - | - | 428 |
| Academic Achievement and Psychosocial Needs of Children Affected or Infected by HIV/AIDS | ASO | 2010 | Youth | - | Other | - | 501 |
| Sports and Social Gay Networks for HIV Prevention | CBO | 2010 | MSM | - | - | Prevention | 247 |
| Elite Controllers for HIV | ASO | 2010 | General HIV+ population | - | - | Epidemiology (i.e. HIV incidence/  prevalence) | 731 |
| Refugees and HIV Prevention | CBO | 2010 | Immigrants/  Refugees/Non-status | - | - | Prevention | 388 |
| Social Media and HIV | ASO | 2010 | General HIV+ population, General HIV- population | - | - | Prevention | 334 |
| Latino Men and Coming Out | CBO | 2010 | MSM, Ethnocultural minorities | Mental health | Social support, Health services, Stigma/discrimination, Other | - | 744 |
| Acceptance Commitment Therapy and its Application in HIV or Stigma Reduction | ASO | 2010 | General HIV+ population | - | Stigma/discrimination | - | 675 |
| HIV Prevention for Men who have Sex with Men | ASO | 2010 | MSM | - | - | Prevention | 537 |
| Provider - Initiated HIV Testing and Counseling | Government Agency | 2010 | Other | - | - | Testing | 567 |
| Transgender Men’s Sexual Health and HIV Risk | Government Agency | 2010 | Transgender communities | - | - | Prevention, Epidemiology (i.e. HIV incidence/  prevalence) | 1081 |
| Coordination and Delivery of HIV Prevention, Treatment, Care and Support by Nurse Practitioners | Hospital/University/Health Clinic | 2010 | Other, General HIV+ population | - | - | Prevention, Treatment/  Adherence | 817 |
| Mandatory testing of Sex Workers for HIV and other Sexually Transmitted Infections | CBO | 2010 | Sex workers | - | - | Testing | 658 |
| Radio-based Interventions for HIV Prevention | CBO | 2010 | General HIV- population | - | - | Prevention | 415 |
| Impact of Housing Status and Supportive Housing on the Health of Aboriginal People | ASO | 2011 | Indigenous communities | - | Housing | - | 576 |
| Psychosocial Support Programs for HIV-Positive Women who are Pregnant | ASO | 2011 | Women | - | Social support | - | 678 |
| Psychosocial Issues for Older Adults Living with HIV/AIDS | ASO | 2011 | Older adults (>50 years) | Mental health | Social support, Health services | - | 553 |
| Models of Outpatient Care for the Ongoing Care and Treatment of People Living with HIV | Government Agency | 2011 | General HIV+ population | - | - | Treatment/  Adherence | 809 |
| Sexual abstinence among people living with HIV/AIDS | ASO | 2011 | General HIV+ population | - | Other | - | 756 |
| Criminalization of HIV exposure or transmission: Stakeholder views and beliefs and effects on behaviour | ASO | 2011 | Other, General HIV+ population | - | Other | - | 385 |
| Special Nutritional Requirements for People Living with HIV/AIDS | ASO | 2011 | General HIV+ population | - | Other | - | 673 |
| Effectiveness and key features of employment support programs | OHTN | 2012 | General HIV+ population | - | Employment | - | 438 |
| East and South East Asian Women’s Sexual Health | ASO | 2012 | Women, Ethnocultural minorities | - | - | Prevention | 1251 |
| Mindfulness - based therapy for people living with HIV | Hospital/University/Health Clinic | 2012 | General HIV+ population | Mental health | - | - | 1036 |
| Sexual health programs for gay and bisexual men in rural and suburban regions | ASO | 2012 | MSM | - | Health services | Prevention | 923 |
| Sex Worker HIV Risk | CBO | 2012 | MSM, Youth, Sex workers | - | Health services | Epidemiology (i.e. HIV incidence/  prevalence) | 4013 |
| Smoking Cessation Interventions in People with HIV/AIDS | OHTN | 2012 | General HIV+ population | Substance use | - | - | 1119 |
| Rapid HIV Testing in Correctional Facilities | Government Agency | 2012 | Prisoners | - | - | Testing | 661 |
| HIV risks associated with tattooing, piercing, scarification and acupuncture | ASO | 2012 | General HIV- population | Co-infections or Co-morbidiities | - | Prevention, Epidemiology (i.e. HIV incidence/  prevalence) | 10353 |
| Demographic characteristics associated with access to HAART, HIV care and HIV viral load testing | ASO | 2012 | General HIV+ population | - | Other | Treatment/  Adherence | 727 |
| Public Perception of Harm Reduction Interventions | Hospital/University/Health Clinic | 2012 | Other, Drug users | Substance use | - | Prevention | 2165 |
| Approaches for Front - Line Organizations to Implement Evidence - Based Interventions | Government Agency | 2012 | General HIV+ population, General HIV- population | - | - | Prevention | 832 |
| Factors influencing the sexual health of Asian men who have sex with men | ASO | 2012 | MSM, Ethnocultural minorities | - | Other | - | 1031 |
| The willingness of PHAs, living in high-income settings to negotiate condom use & use condoms during sex | OHTN | 2013 | General HIV+ population | - | - | Prevention | 521 |
| Complementary, Alternative and Traditional Medicine in HIV Care | ASO | 2013 | Ethnocultural minorities, General HIV+ population | - | - | Treatment/  Adherence | 4187 |
| Effects of Viral Load and Antiretroviral Medications on Sexual Transmission of HIV | Government Agency | 2013 | General HIV+ population | - | - | Epidemiology (i.e. HIV incidence/  prevalence), Treatment/  Adherence | 2020 |
| Intersectionality in HIV and Other Health-Related Research | ASO | 2013 | General HIV+ population | - | Other | - | 2096 |
| Gay Men’s Attitudes and Perceptions Regarding Rapid HIV Home Testing | OHTN | 2013 | MSM | - | - | Testing | 1177 |
| Migrant Farm Workers and Sexual Health | ASO | 2013 | Immigrants/Refugees/Non-status, Other | - | Health services, Other | - | 1518 |
| Effectiveness of HIV Testing Interventions for High-Risk Populations | Government Agency | 2013 | MSM | - | - | Testing | 1159 |
| Adherence to Methadone Maintenance Treatment and Antiretroviral Therapy | Hospital/University/Health Clinic | 2013 | Drug users, General HIV+ population | Substance use | - | Treatment/  Adherence | 1490 |
| HIV Services in Rural and Remote Communities | ASO | 2013 | Other | - | Health services, Stigma/discrimination | - | 1486 |
| Female condom use among MSM | Government Agency | 2013 | MSM | - | - | Prevention | 2896 |
| How to support and facilitate peer engagement in service provision roles | ASO | 2013 | General HIV+ population | - | Health services | - | 3024 |
| Gay men’s attitudes and perceptions regarding PrEP and PEP | OHTN | 2013 | MSM | - | - | Prevention | 1773 |
| Effective HIV prevention, education and outreach activities in African, Caribbean and Black communities | ASO | 2014 | Ethnocultural minorities | - | Education | Prevention | 1934 |
| Gay men’s attitudes and perceptions regarding viral load and treatment as prevention | OHTN | 2014 | MSM | - | - | Prevention, Treatment/  Adherence | 572 |
| Facilitators and barriers to health care for lesbian, gay and bisexual (LGB) people | OHTN | 2014 | MSM, Other | - | Health services | - | 6084 |
| HIV prevalence and testing among street-involved youth in Ontario | Government Agency | 2014 | Youth | - | - | Epidemiology (i.e. HIV incidence/  prevalence), Testing | 1121 |
| Online counseling and support groups for people living with HIV | ASO | 2014 | General HIV+ population | - | Social support | - | 2373 |
| Common conditions and diseases in HIV-positive men who have sex with men | Hospital/University/Health Clinic | 2014 | MSM | Substance use, Co-infections or Co-morbidiities | - | - | 1102 |
| Peer-based programs to support antiretroviral adherence | ASO | 2014 | General HIV+ population | - | Health services | Treatment/  Adherence | 989 |
| Case management/community development models | ASO | 2014 | Other | Substance use, Mental health, Co-infections or Co-morbidiities | Health services | - | 1121 |
| Behavioural emergencies among HIV positive gay adult men | Hospital/University/Health Clinic | 2014 | MSM | Mental health | - | - | 582 |
| Effectiveness of motivational interviewing in changing risk behaviours for people living with HIV | OHTN | 2013 (Updated 2014) | General HIV+ population | - | Health services | Prevention | 1266 |
| What is the effectiveness of supervised injection services? | ASO | 2014 | Drug users | Co-infections or Co-morbidiities | - | Prevention | 6562 |
| Disclosure of HIV-positive status: Towards the development of guidelines, strategies and interventions | Government Agency | 2013 (Updated 2014) | General HIV+ population | - | Social support, Stigma/discrimination | - | 2864 |
| Telemedicine and HIV Health Care | OHTN | 2014 | General HIV+ population | - | Health services | Treatment/  Adherence | 718 |
| Hepatitis C virus (HCV) reinfection rates among people who use drugs | Government Agency | 2014 | Drug users | - | - | Epidemiology (i.e. HIV incidence/  prevalence) | 1316 |
| Treatment access barriers and related best practices for newcomers living with HIV and/or HCV | ASO | 2014 | Immigrants/Refugees/Non-status | - | Health services, Stigma/discrimination, Other | Treatment/  Adherence | 771 |
| Posttraumatic stress disorders among people living with HIV/AIDS | ASO | 2014 | General HIV+ population | Mental health | -- |  | 594 |
| Sexual health of heterosexually-identified men who have sex with men | ASO | 2014 | MSM | - | - | Prevention, Testing | 668 |
| Crystal methamphetamine use, sexual risk behaviours and harm reduction among men who have sex with men | ASO | 2015 | MSM | Substance use | - | Prevention | 1042 |
| HIV-related stigma in relation to health care professionals in Canada | ASO | 2015 | Other | - | Stigma/discrimination | - | 560 |
| Reminder systems for people living with HIV | Government Agency | 2015 | General HIV+ population | - | - | Epidemiology (i.e. HIV incidence/  prevalence), Testing, Retention in care, Treatment/  Adherence | 303 |
| Transitioning from Adolescent to Adult Care in HIV | OHTN | 2015 | Youth | - | - | Linkage/  engagement in care | 621 |
| Impact of Community-Based Organizations for People Living with HIV | ASO | 2015 | General HIV+ population | - | Social support, Health services | Testing, Treatment/  Adherence | 436 |
| Knowledge of HIV and Related Best Practices Among Non-HIV Specific Health Care Providers | ASO | 2015 | Other | - | Health services | - | 408 |
| Mobile Apps and Sexual Risk Behaviors among Men who Have Sex with Men | CBO | 2015 | MSM | - | - | Prevention | 3866 |
| Factors affecting the health and well-being of lesbian, gay and bisexual Asian youth | ASO | 2015 | MSM, Youth, Ethnocultural minorities | - | - | - | 533 |
| Epidemiology of hepatitis C virus infection among men who have sex with men | ASO | 2016 | MSM | Co-infections or Co-morbidiities | - | - | 542 |
| The role of nurse practitioners in HIV care | Hospital/University/Health Clinic | 2016 | Other | - | Health services | - | 718 |
| Delivering HIV/STI test results over the phone and through text messaging | Government Agency | 2016 | General HIV+ population | - | Health services | - | 674 |
| Programs to improve the sexual health and well-being of transgender individuals | ASO | 2016 | Transgender communities | - | Health services | - | 596 |
| HIV and STI testing among Indigenous women and women who inject drugs | ASO | 2016 | Drug users, Indigenous communities | Substance use | - | Testing | 465 |
| Hepatitis C reinfection after successful treatment | ASO | 2016 | General HIV+ population, General HIV- population | Co-infections or Co-morbidiities | - | Treatment/  Adherence | 1148 |
| Impact of successful hepatitis C treatment on quality of life | ASO | 2016 | General HIV+ population, General HIV- population | Co-infections or Co-morbidiities | Health services | - | 657 |
| Chest compressions and/or the use of rescue breathing when responding to an opioid overdose and administering naloxone | Government Agency | 2016 | Drug users | Substance use | - | - | 274 |
| Engaging law enforcement in harm reduction programs | Government Agency | 2016 | General HIV- population | Substance use | - | Prevention | Not available |
| The impact of mental health issues on ART adherence, sexual risk behavior and overall health in MSM | Government Agency | 2016 | MSM | Mental health | - | Treatment/  Adherence | 289 |
| Improving client/patient health literacy | OHTN | 2016 | Other, General HIV+ population | - | Health services | - | 238 |
